# Feasibility of Adding Enhanced Pedometer Feedback to Nutritional Counseling for Weight Loss

**DOI:** 10.2196/jmir.7.5.e56

**Published:** 2005-11-17

**Authors:** Caroline R Richardson, Beverley B Brown, Sharon Foley, Kathleen S Dial, Julie C Lowery

**Affiliations:** ^4^Veterans Affairs Medical CenterTucsonAZUSA; ^3^Veterans Affairs Medical CenterChicagoILUSA; ^2^University of Pittsburgh Medical CenterPittsburghPAUSA; ^1^Veterans Affairs Medical Center and University of Michigan Health SystemsDepartment of Family MedicineAnn ArborMIUSA

**Keywords:** obesity, diet, exercise, cardiovascular diseases, risk factors, Internet

## Abstract

**Background:**

Intensive interventions targeting diet and physical activity are effective for weight reduction but are costly. Tailored, computer-generated, step-count feedback may provide an intensive and affordable way to increase the physical activity of people at high risk for cardiovascular disease.

**Objective:**

The objective was to test the feasibility of adding tailored, computer-generated, step-count feedback to a face-to-face nutritional counseling weight loss intervention.

**Methods:**

We recruited 12 participants, 4 from each of three Department of Veterans Affairs medical centers. There were 11 male participants and 1 female participant. Each had a body mass index of 30 or greater and at least one of the following cardiovascular disease risk factors: diabetes, hypertension, hypercholesterolemia, obesity, or coronary artery disease. Participants attended one-on-one counseling sessions with a registered dietitian for four sessions over three weeks. At the initial session, each participant received an enhanced pedometer to record time-stamped, step-count data. Participants wore the device daily throughout the intervention. At the three follow-up sessions, the dietitian uploaded the computer data, reviewed a Web-based graphical display of step-count feedback, and helped set new walking goals.

**Results:**

All 12 participants completed the program (100% attendance). Initial mean weight was 255 lbs (SD = 49 lbs), and weight loss was just over 4 lbs (n = 12, paired *t* test, *P* = .004). Mean daily step counts during the first week averaged 6019 steps per day, increasing to an average of 7358 per day after the third week (average increase of 1339 steps per day, or 0.6 miles, or 12 minutes of walking, n = 10, paired *t* test, *P* = .04).

**Conclusions:**

Enhanced pedometer feedback in conjunction with nutritional counseling is feasible and results in significant weight loss and increased walking among individuals at high risk for cardiovascular disease.

## Introduction

Being sedentary and eating a poor diet increases the risk of developing diabetes [1-3], heart disease [4-8], hypertension [9], depression [10-12], and many other chronic diseases [13]. We now know that with intensive monitoring, counseling, and encouragement, individuals can change their diet and exercise behaviors and that such changes result in dramatic improvements in health outcomes. In the Diabetes Prevention Program study, over 1000 individuals at high risk for developing diabetes who were randomized to an intensive diet and physical activity intervention decreased their risk of developing diabetes by almost 60% over three years compared to individuals in the usual care arm [2]. Unfortunately, brief clinical interventions designed to help patients change their diet and physical activity have not been effective [14]. The challenge is to find cost-effective and feasible ways to integrate intensive diet and exercise interventions into clinical practice.

One approach to decreasing the cost and increasing the intensity of lifestyle interventions is to automate the process of diet and activity counseling using tailored Internet-based systems. While such systems have shown promise, low participant adherence in terms of returning to the website for reinforcement has proven to be a major barrier [15-18]. Automated systems may work better to augment existing relationships with health care providers rather than replacing the health care provider all together.

Behavioral researchers have shown that people have poor recall of daily walking duration, intensity, and frequency [19]. This poor recall makes it difficult to set short-term, incremental behavioral goals or to reward successes [16,20]. Clear and objective feedback about daily walking patterns can be useful in overcoming barriers, setting goals, and rewarding successes. Pedometers are devices worn on the waist or belt that count steps taken throughout the day. Walking interventions that use pedometers and specific step-count goals are more effective than walking interventions that use time-based goals. Hultquist et al found that sedentary women who were given pedometers and who were instructed to walk 10000 steps a day walked almost 2000 steps per day more than women who were instructed to go for a brisk 30-minute walk each day [21].

Unlike simple pedometers that keep a single running total of step counts, enhanced pedometers record detailed, time-stamped, step-count data. Once these data are uploaded to a computer, they can be used to automatically generate individually tailored reports about the duration and intensity of walking bouts throughout the day. Users can then review the tailored step-count feedback in graphical or numerical form.

In this study, we examine the feasibility of integrating an Internet-based enhanced pedometer system into routine nutritional counseling for individuals with cardiovascular disease (CVD) risk factors. Our primary goal was to assess health service delivery parameters, including required face-to-face counseling time, participant satisfaction, and technical barriers to delivering this type of intervention. We also assessed weight loss and increased walking among the participants.

## Methods

### Study Design

This was a multi-center intervention feasibility study with a pre-post design. The intervention consisted of four individual face-to-face nutritional counseling sessions for weight loss over a three-week period. Dietitians incorporated computer-generated, tailored, step-count feedback and goal setting to increase walking into each of the four visits. We recruited 12 participants, 4 from each of three different Veterans Affairs (VA) medical centers to assess portability and feasibility of the intervention. This study was approved by the Investigational Review Boards at the participating sites and by the Ann Arbor Veterans Affairs Medical Center. All eligible participants signed written informed consent documents prior to enrollment.

### Study Population

Participants were adult VA patients who were referred by a VA physician for outpatient nutritional counseling. Dietitians at each site offered patients the opportunity to participate if they were ambulatory, sedentary, overweight (body mass index [BMI] > 28), and were at high risk for CVD. Patients were considered ambulatory if they could comfortably walk one block. Sedentary was defined as less than 150 minutes of physical activity of at least moderate intensity each week by self-report. Individuals were considered at high CVD risk if they had one of the following diagnoses: (1) type 2 diabetes, (2) hypertension, (3) hypercholesterolemia, (4) obesity (BMI > 30), or (5) known coronary artery disease. CVD risk status was assessed by self-report and confirmed by chart review. Smoking was not included in this list because there is relatively little evidence that physical activity reduces CVD risk in smokers who have no other CVD risk factors. All participants were either in the contemplation or preparation stage for starting a walking program [22,23].

Participants were not required to get medical clearance to participate in this study. Because the study emphasized a gradual increase in walking at a moderate pace, the risk of adverse cardiac events while walking was low. However, participants who reported chest pain, shortness of breath, or lightheadedness while walking, who had been advised by a physician not to walk, or who were undergoing a cardiac work-up were excluded from the study. Individuals who had been enrolled in another nutritional counseling program within the past 28 days were excluded. Participants were also excluded if they could not comfortably communicate in English or if they were not competent to give informed consent.

### The Pedometer

**Figure 1:**
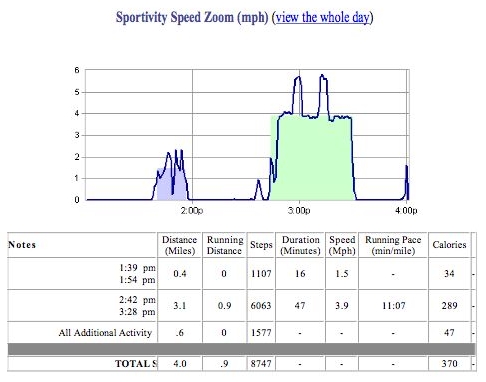
Sample graph and table from the SportBrain website

**Figure 2:**
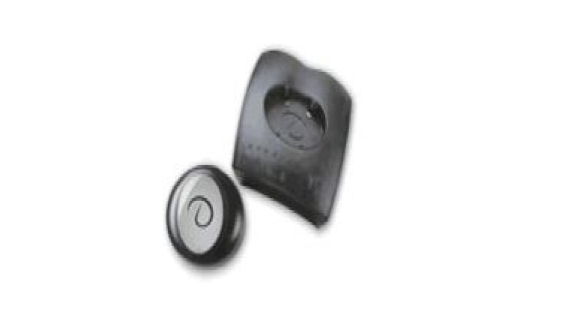
The SportBrain First Step enhanced pedometer and Sportport interface

All participants were given a SportBrain First Step enhanced pedometer (www.sportbrain.com) to wear throughout the intervention period. The device we used uploaded step counts over a phone line to a central computer. The SportBrain website then displayed individually tailored graphs of step counts along with motivational messages. Participants wore the pedometer every day, all day, for approximately 22 days per participant (4 weekly sessions). While more recent versions of the enhanced pedometers display the total daily step count on the pedometer itself, the First Step device used in this trial did not have a display on the pedometer. The only way to review the step-count feedback was to upload it to the computer and review it online. Figure 1 shows a sample of the Internet-based feedback provided on the SportBrain website. A wide variety of graphs are available on the website. The graph in Figure 1 shows time on the x axis and miles per hour on the y axis for a period of high activity on one day. Time stamping in the step-count data allows estimation of physical activity intensity and duration of activity bouts. Figure 2 shows the SportBrain pedometer and upload dock that we used in this intervention.

### Intervention

Each participant met with a dietitian during four individual face-to-face sessions for both diet and physical activity counseling. The diet intervention was a structured nutritional counseling intervention based on the American Dietetic Association’s Medical Nutrition Therapy Weight Management Protocol [24]. This is a detailed six-session program, which we modified to a more feasible four-session protocol.

During the first session, which lasted approximately one hour, the study was described, and patients who agreed to participate and met eligibility criteria completed a written informed consent process. Participants were given a SportBrain First Step pedometer and were taught how to position the pedometer on their waistband, attach the safety clip to their belt or clothes, and check to see if the pedometer was counting steps properly using a 20-step test walk. Participants also completed a brief nutritional assessment and received preliminary nutritional counseling.

During visits 2 through 4, participants met individually face-to-face with their study dietitian for both nutritional counseling and reinforcement of the walking program. Participants reviewed step-count data on the computer with the dietitian and negotiated new step-count goals for the upcoming week. Step-count goals were increased gradually. For example, a participant who averaged 3000 steps a day during week one might have set a new goal of 3500 steps per day for week two. Dietitians then discussed nutritional counseling topics using standardized handouts and set new nutritional goals for the week. Finally, participants filled out a brief questionnaire about attitudes toward the Internet-based step-count feedback, problems using the enhanced pedometer, and self-efficacy for achieving walking goals. Participants received US$10 for each of the four visits attended. At the end of the session, participants who returned the enhanced pedometers received a “Veterans Walk for Health” T-shirt valued at US$10.

### Measures

#### Feasibility Outcomes

The following components of feasibility were measured explicitly:

1. **Participant satisfaction** with the intervention as measured by responses to a brief satisfaction survey. This survey included questions about overall satisfaction with the intervention and about willingness to pay to continue to have access to the intervention after the study was over. Finally, participants were asked what they liked and did not like about the enhanced pedometer component of the intervention in a series of open-ended questions.
2. **Participant adherence** to the intervention was assessed using three measures: (1) attendance at visits, (2) self-report of the number of study days that pedometer was worn, and (3) electronic step-count data indicating days that the pedometer was worn.
3. **Counseling time:** Mean face-to-face dietitian counseling time required to deliver the physical activity and nutritional counseling components of the intervention was recorded at each visit by the study dietitian on a study visit data collection form.
4. **Technical problems** with uploading the pedometer data to the website were logged by the study dietitian on the study visit data collection form at each visit.

#### Intervention Outcomes

1. **Participant weight:** A single clinical scale at each site was designated as the study scale. Scales were zeroed before each participant was weighed. Participants were weighed without shoes, and weight was recorded at each visit.
2. **Mean daily step count:** During the final week of the intervention, this was recorded electronically by uploading the data to the website.
3. **Participant self-efficacy** for adhering to a walking program as measured by participants' response to a single question: "I am confident that I can reach my new step-count goals next week." (possible responses were "Strongly Disagree", "Disagree", "Neutral", "Agree", and "Strongly Agree").

Training for the dietitians who participated in the study took place in two phases. After an introductory conference call, all participating dietitians wore the pedometer for one week, uploaded their own walking data at least twice during this trial period, and learned to navigate around the SportBrain website by viewing different formats for step-count feedback and changing their step-count goals. After the trial period, we had a series of conference calls to discuss and troubleshoot technical problems with the devices and to review counseling guidelines.

### Sample Size

We did not power this feasibility study to be able to detect clinically significant weight loss or increase in average daily step counts. Instead, we intentionally kept the overall sample size small but recruited from three different VA medical centers that were remote from the coordinating center. Recruiting from multiple sites increased the complexity of the study in that we were required to get human subjects committee approval from four different medical centers, and we had to train six different dietitians. However, recruiting from multiple sites increased the power to examine potential barriers to implementation and feasibility issues across multiple settings.

### Data Analysis

Means and standard deviations were used to estimate time spent in both the step-count and the nutritional counseling components of the intervention. Mean participant satisfaction and percentage of sessions attended were also calculated. For pre-post comparisons, we used paired *t* tests. We compared mean daily step counts for the first 7 days of the intervention with mean daily step counts for the final 7 days of the intervention. Similarly, paired *t* tests were used to test for statistically significant weight loss.

We anticipated that, as the participant became more familiar with the pedometer and with the nutritional counseling component of the intervention, the time required for counseling might decrease. To test this hypothesis, we compared the counseling time difference between the second and fourth visits. We did not use the initial session for time comparison because of the substantial time required to enroll the participant in the study and explain the intervention. Finally, participant comments in response to open-ended questions about the intervention were tabulated and reviewed for common themes. All statistical analyses were performed using Stata version 8 [25].

## Results

### Quantitative Results

From September to December 2003, a total of 12 participants (11 male and 1 female) were enrolled from three different VA hospitals. Recruitment of four eligible and interested participants was completed within one month at each of the three sites for an average recruitment rate of one participant per week per site. Though obesity was not a criterion for enrollment, all 12 participants had a BMI greater than 30. Participants weighed an average of 255 pounds (SD = 49) at baseline. BMI averaged 37 (SD = 6.5). Ten of the 12 participants also had at least one other CVD risk factor in addition to their obesity. The participants were followed for an average of 21 days (range 18-27 days), including the initial day and last day. Table 1 provides details on the participants’ baseline characteristics.

**Table 1:** Participant baseline characteristics (N = 12)

**Chronic Conditions**		**N (%)**
	Diabetes	2 (17%)
	Hypertension	8 (67%)
	Hypercholesterolemia	6 (50%)
	Obesity	12 (100%)
	Coronary artery disease	1 (8%)
		**Mean**+**SD**
Height (inches)		69.4 + 2.5
Baseline weight (pounds)		254.9 + 49.4
Body mass index (kg/m^2^)		37 + 6.5
Age (years)		52.7
		**Mean**+**SD or (%)**
**Comfort with Computers** (1 = not; 4 = very)		3.6 + 0.5
	1	0
	2	0
	3	5 (41.7%)
	4	7 (58.3%)
**Initial Self-Efficacy to Increase Walking** (1 = not; 5 = very)		4.5 + 0.7
	1	0
	2	0
	3	1 (8.3%)
	4	4 (33.3%)
	5	7 (58.3%)

All 12 pedometers were returned in working condition at the end of the study. Of the 36 attempts to upload step-count data (12 participants times 3 sessions), 32 were successful. During 4 (11%) of the sessions, there were problems with uploading the data. Two of the upload attempts failed for administrative reasons related to access to the website, and one failed because the Internet connection was down. The remaining session failed because the pedometer was not recording steps properly.

Table 2 provides participant satisfaction measures as well as face-to-face time per visit. All 12 participants attended each of the four scheduled study visits for a 100% attendance rate and a 100% follow-up rate. Electronic monitoring data show that participants wore the pedometer for more days than they could recall. Participants reported remembering to wear the pedometer for the whole day for 95% (243 of 257) of patient days in the study. According to electronically recorded step-count data, the pedometers were worn at least part of the day for 99% (254 of 257) of patient days of the study.

Overall participant satisfaction at the final visit averaged 4.4 out of 5 on a 5-point scale, in which 5 indicated very satisfied; 11 out of 12 were very satisfied or satisfied. Satisfaction was also assessed by asking participants if they would be willing to pay for the enhanced pedometer. The cost participants were willing to assume ranged from US$0 to $100 per year. The average price a veteran was willing to pay for a year of pedometer service was US$20.50. The average total time per face-to-face counseling session was 33 minutes.

**Table 2:** Main results

		**Mean**+**SD or (%)**
Willingness to pay for service (US$)		20.50 + 30.8
Nutritional counselling session attendance, 4 total		4 + 0
**Participant satisfaction with intervention** (1 = very unsatisfied; 5 = very satisfied)		
	1	1 (8.3%)
	2	0
	3	0
	4	3 (25.0%)
	5	8 (66.7%)
**Post-Intervention Self-Efficacy to Increase Walking**(1 = not; 5 = very)		
	1	0
	2	0
	3	0
	4	8 (66.7%)
	5	4 (33.3%)
**Counseling time for nutrition (min)**		
	Visit 1 (initial set-up)	19.49 + 12.15
	Visit 2	21.25 + 10.47
	Visit 3	14.00 + 6.99
	Visit 4	13.64 + 8.97
**Counseling time for step count (min)**		
	Visit 1 (initial set-up)	13.92 + 11.85
	Visit 2	16.82 + 9.02
	Visit 3	18.64 + 8.09
	Visit 4	14.09 + 6.64
**Total counseling time for both (min)**		
	Visit 1 (initial set-up)	33.41
	Visit 2	38.07
	Visit 3	32.64
	Visit 4	27.73

The average weight loss was 4.1 pounds over three weeks (n = 12, paired *t* test, *P* = .004); 11 out of 12 participants lost weight. Weight loss ranged from –1.8 pounds to 9.7 pounds. Only one of the 12 participants gained weight. By the last week of the trial, participants significantly increased daily step counts. Mean daily step counts during the first week averaged 6019 steps per day, increasing to an average of 7358 per day after the third week (average increase of 1339 steps per day, n = 10, paired *t* test, *P* = .04). This increase translates into a daily increase of 0.6 miles, or 12 minutes of walking assuming that 2200 steps is a mile and that the average walking pace is 3 miles per hour. There was no significant change in self-efficacy between the first and final session. There were no major adverse events and, in particular, no adverse cardiovascular events during the intervention.

### Qualitative Results

The most common problem with the pedometer was difficulty attaching it to the waistband or belt. Participants liked viewing graphs of step counts because it documented their successes and made it easy to see step-count targets or goals. One participant commented that “it is a new way to look at my walks…” and another liked “knowing what [he] accomplished for the week.” Others liked seeing the relationship between steps taken and calories burned, which is printed in a table on the website. Participants also liked being able to see the number of steps taken during a particular activity, such as on a specific walk or while shopping. In general, the participants did not like seeing that they were not doing enough walking to meet their step-count goals. Even participants who regularly met their step-count goals were worried about how they would feel if they failed.

## Discussion

Our study demonstrates the feasibility of incorporating enhanced pedometers and Internet-based feedback into traditional nutritional counseling programs to promote weight loss. We were able to implement the enhanced pedometer intervention in three different medical centers with minimal training and technical support. While participants did have some trouble wearing the pedometer, they did not have trouble understanding the step-count feedback graphs, and satisfaction with the intervention was high. While we did not expect to see significant changes in either weight or step counts in this small and brief feasibility study, results for weight loss and step-count increases were both statistically and clinically significant.

Several recent studies have examined the effectiveness of Internet-based weight loss programs without face-to-face counseling [18,26,27]. The interventions tested in these studies have relied primarily on self-reported diet and physical activity logs and text-based behavioral counseling. Our intervention differs from these previously tested Internet-based weight loss programs in several ways. First, the enhanced pedometer and Internet-based, step-count feedback were used as an intensive and objective monitoring tool and not as a substitute for one-on-one counseling. By automating the process of uploading step-count data, enhanced pedometers reduce respondent burden involved in filling out logs and also increase the detail and accuracy of the data. Secondly, our intervention had a balanced approach, focusing on both diet and physical activity in approximately equal parts. While the exact role of physical activity in initial weight loss is still being debated, there is a consensus that physical activity is critical for long-term weight control [28-30].

The theory of self-regulation suggests that helping individuals learn how to more accurately and more intensively self-monitor the behavior they are trying to change should result in improved outcomes [16,20]. At least one study has shown that consistent self-monitoring of diet leads to better weight loss and control [33]. Similarly, success with simple pedometer interventions suggests that objective monitoring and step-count feedback can increase walking [21,34,35]. Using the existing information technology to optimize self-monitoring of both physical activity and diet in combination with one-on-one counseling, delivered either face-to-face, by telephone, or by email, may prove to be a cost-effective intervention for initially reducing weight and for subsequently maintaining weight loss. Detailed step-count feedback may augment the effect of face-to-face counseling sessions and may reduce the face-to-face counseling time required for effective behavior change interventions. More research will be needed to determine the optimal frequency and intensity of self-monitoring, group sessions, and one-on-one contact to maximize long-term maintenance of weight loss while keeping costs reasonable.

The cost of enhanced pedometer systems is dropping rapidly. When this feasibility study was conducted, the cost of a SportBrain enhanced pedometer, a Sportport computer interface dock, and a year-long subscription to the website was about US$200. Currently, the new and improved version of the SportBrain enhanced pedometer can be purchased for US$30 or less, a device-specific interface port is no longer needed, and basic Web access is free. There are a number of other companies on the market that are producing enhanced pedometers, and the cost is likely to continue to drop.

Limitations to this study include the small sample size, the short duration of the intervention, and the lack of a control group. Despite the small sample size and short duration, we did find significant reductions in weight and increases in walking. In addition, this study used enhanced pedometers with nutritional counseling for all participants, and hence, we are unable to estimate the component of weight loss that was attributable to the enhanced pedometer system alone.

There are many advantages to having detailed time-stamped data uploaded to a central computer rather than relying on the participant to log daily step counts on a paper calendar or type them into a Web page. The decreased respondent burden, the ability to automate the creation of tailored motivational messages based on previous step counts, as well as the ability to examine detailed walking patterns throughout the day, including duration and intensity data, may make enhanced pedometers well worth the small increase in cost over simple pedometers. The benefits of using an enhanced pedometer will depend on our ability to create sophisticated, reliable, and user-friendly, Web-based systems for clinical interventions. This approach may be best suited to a health care system using a team approach of dietitian, patient, and physician combined with Internet monitoring. Creating disease-specific walking websites that give enhanced pedometer feedback or websites that can tailor motivational messages and set goals based on current disease state may further increase the value of such interventions [36-38].

Advances in information technology have yielded low-cost, user-friendly, intensive physical activity monitoring and feedback systems. Results of this study demonstrate that enhanced pedometers with Web-based, step-count feedback can be integrated into a nutritional counseling program focusing on weight loss. This intervention is portable as it was successfully implemented at three different medical centers. Participants lost weight and increased their daily walking during the brief intervention. Further research and randomized controlled trials with assessment of long-term outcomes are needed to test the impact of incorporating enhanced pedometer step-count feedback into routine medical care for high-risk individuals.
